# Clinical Features of Anti-Synthetase Syndrome Associated with Prognosis in Patients with Dermatomyositis and Polymyositis

**DOI:** 10.3390/jcm11072052

**Published:** 2022-04-06

**Authors:** Sung Soo Ahn, Yong-Beom Park, Sang-Won Lee

**Affiliations:** 1 Department of Internal Medicine, Yongin Severance Hospital, College of Medicine, Yonsei University, Yongin 16995, Korea; saneth@yuhs.ac; 2 Division of Rheumatology, Department of Internal Medicine, College of Medicine, Yonsei University, Seoul 03722, Korea; yongbpark@yuhs.ac; 3 Institute for Immunology and Immunological Diseases, College of Medicine, Yonsei University, Seoul 03722, Korea

**Keywords:** dermatomyositis, polymyositis, mortality, fever, anti-synthetase syndrome, clinical features

## Abstract

We evaluated whether the clinical features of anti-synthetase syndrome (ASA)—myositis, fever, arthritis, mechanic’s hand, Raynaud’s phenomenon and interstitial lung disease—are relevant to prognosis in patients with dermatomyositis/polymyositis (DM/PM). A retrospective analysis was performed to identify patients diagnosed with DM/PM according to Bohan and Peter criteria. Clinical information, laboratory data and the presence of ASA clinical features at disease diagnosis were searched, and the outcomes of all-cause mortality, intensive care unit admission and disease remission at 1 year were assessed. Among the 86 patients included, fever (36.0%) and interstitial lung disease (26.7%) were the most common ASA clinical features. During the follow-up, 12 patients experienced death, and 7 of the 12 deaths (58.3%) occurred within 3 months of DM/PM diagnosis. Mortality was more frequently observed in those presenting with fever than in those without (25.8% versus 7.3%, *p* = 0.024). Multivariable Cox proportional analysis revealed that male sex (hazard ratio [HR] 5.53, 95% confidence interval [CI] 1.65, 18.49, *p* < 0.01) and fever (HR 4.20, 95% CI 1.26, 14.01, *p* = 0.02) independently predicted mortality. The clinical impact of fever was consistent in both sexes. Fever could be a warning signal heralding the poor outcome of mortality in patients with DM/PM, especially in early disease phases.

## 1. Introduction

Anti-synthetase syndrome (ASA) refers to a heterogeneous group of systemic autoinflammatory disorders (AIDs) associated with antibodies against aminoacyl-transfer RNA synthetases (ARS) [1]. It is suggested that ASA, which was first recognised in the 1990s, is a clinical entity that could present with a constellation of phenotypes including myositis, fever, arthritis, mechanic’s hand, Raynaud’s phenomenon, and interstitial lung disease (ILD) [1,2,3]. Of note, clinical features of ASA could be present in patients with various immune-mediated rheumatic diseases, and studies indicate that these features of ASA are associated with patient prognosis. In rheumatoid arthritis, which is one of the most common AIDs of the joints, the presence of ILD has been reported to be associated with increased mortality [4]. Similarly, ILD has a significant impact on mortality in patients with systemic lupus erythematosus (SLE) and mixed connective tissue disease [5]. Furthermore, studies have suggested that ILD is related to a worse prognosis in patients with anti-neutrophil cytoplasmic antibody-associated vasculitis [6]. Moreover, fever, especially the complication of macrophage activation syndrome, is a well-known poor prognostic factor in systemic juvenile idiopathic arthritis and has been reported to be associated with adverse prognosis in patients with SLE [7,8,9]. Taken together, it could be hypothesised that patients with AIDs could have a different disease course depending on the presence of ASA clinical features.

Idiopathic inflammatory myopathies (IIM) are AIDs that primarily affect the muscles (myositis) and show a difference in clinical presentation, laboratory test results, and imaging and electromyographic findings [10]. Dermatomyositis (DM), polymyositis (PM), necrotising autoimmune myositis, and inclusion-body myositis are four representative diseases comprising IIM, and DM and PM are the most widely recognised forms of myopathies. Typically, DM can affect adults and children and is common in female individuals with characteristic dermatologic manifestations—i.e., heliotrope rash, Gottron papule, shawl sign, and V-sign—whereas PM does not cause skin lesions, usually affects female individuals older than 20 years of age and is rare in childhood [11]. Although DM and PM are uncommon, they are now being increasingly understood as potential life-threatening diseases requiring optimal treatment and the identification of factors associated with mortality. A nationwide analysis by Airio et al. showed that patients with DM/PM were three times more likely to die than the general population, and a previous study revealed that long-term mortality associated with DM/PM exceeds 10% [12,13]. In addition, a study by Marie et al. showed that the overall mortality of DM/PM reached 22% [14]. Consistent with the observation of Marie et al., Yang et al. suggested that 21.9% of patients in a cohort with DM/PM died, and comparable five-year survival estimates were found in a study from the United States [15,16]. In general, conventional risk factors associated with worse prognosis in patients with DM/PM are older age, male sex, involvement of major organs, malignancies, and the presence of myositis-specific antibodies [17]. However, owing to the wide variability of study designs and clinical and laboratory features of affected patients and the general population, the relevant risk factors of these diseases are not well established, and inconsistencies have been reported between studies. Besides, while it has been described that clinical features of ASA could be present in DM/PM, it is unclear whether these features could affect patient prognosis. To this end, the purpose of this study was to examine the prognostic implications of these features in patients with DM/PM by reviewing the medical records of patients diagnosed as having DM/PM.

## 2. Materials and Methods

### 2.1. Study Design and Patient Selection

**Figure 1:**
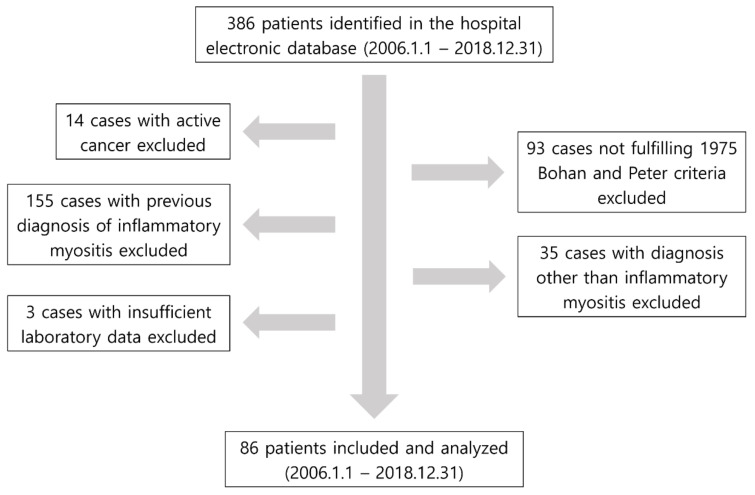
A schematic figure of patient selection.

We performed a retrospective analysis to review the electronic medical records of patients diagnosed as having DM and PM between January 2006 and December 2018 in two university-affiliated Severance Hospitals located in Sinchon and Gangnam, Republic of Korea. For patient selection, we first identified 386 patients on the basis of the International Classification of Diseases and the Tenth Revision codes of DM and PM (either suspected or established diagnosis), utilising the Clinical Data Retrieval System of our hospital. Next, we excluded 297 patients according to the following exclusion criteria: (i) patients who did not fulfil the definite 1975 Bohan and Peter criteria [18,19]; (ii) those who were previously diagnosed as having DM/PM; (iii) those who had concomitant malignancies, as myositis could be a manifestation of paraneoplastic syndrome; (iv) those with other connective tissue diseases other than DM/PM. In addition, three patients whose laboratory results were not available were excluded. As a result, 86 patients were included in the present study (Figure 1). The Institutional Review Board of Yongin Severance Hospital approved the present study, and as this study was conducted retrospectively, the requirement for written informed consent was waivered (9-2020-0156).

### 2.2. Collection of Baseline Patient Data

For baseline patient data, age at diagnosis, patient’s sex, final diagnosis, and previous comorbidities of hypertension, diabetes mellitus, and dyslipidaemia prior to disease diagnosis were recorded. Laboratory data consisted of white blood cell, neutrophil, and platelet counts, erythrocyte sedimentation rate (ESR), C-reactive protein level, aspartate aminotransferase, alanine aminotransferase, creatinine kinase, and anti-Jo-1 antibody positivity at DM/PM diagnosis.

### 2.3. Clinical Features of Anti-Synthetase Syndrome, Definition of Clinical Outcomes and Treatment

The presence of ASA clinical features—fever ≥37.8 °C confirmed on hospital visit or admission [8], Raynaud’s phenomenon, mechanic’s hand, arthritis, and ILD documented by a radiographic study of chest x-ray and/or chest computed tomography—at disease diagnosis was determined by reviewing the medical records of the eligible patients. In patients with fever, the possibility of concomitant infection was excluded based on the findings of physical examination and additional blood and radiographic tests.

Three clinical outcomes including all-cause mortality, intensive care unit (ICU) admission, and disease remission at 1 year were evaluated. Remission at 1 year was defined as non-detectable clinical and biochemical activity attributed to DM/PM, with a modification from a previous study [12]. The follow-up duration for patients experiencing death or ICU care was assessed by calculating the time interval from disease diagnosis to the first occurrence of the corresponding event, whereas it was estimated from disease diagnosis to the last hospital visit for those who did not die or were not admitted to the ICU. The last follow-up date was in January 2019.

Medications including glucocorticoids, methotrexate, azathioprine, intravenous immunoglobulin, rituximab, hydroxychloroquine, cyclophosphamide, tacrolimus, and mycophenolate mofetil that were administered to the patients after disease diagnosis to the last follow-up for the purpose of disease control were recorded.

### 2.4. Statistical Analysis

All statistical analyses were performed using MedCalc statistical software version 19.6 (MedCalc Software, Ostend, Belgium). Continuous variables are expressed as median and interquartile range, and categorical variables as frequency and percentage. The difference between categorical variables was estimated using the chi-square test or the Fisher’s exact test, as indicated. Kaplan–Meier’s analysis with the log-rank test was used to compare the clinical outcomes of patients according to the presence of fever. Univariable and multivariable Cox proportional hazard analyses with the forward entry method was performed using variables with clinical significance in univariable analysis to investigate predictive factors of mortality. In all analyses, a two-tailed *p*-value < 0.05 was considered statistically significant.

## 3. Results

### 3.1. Patient Characteristics

The median age of the 86 patients was 51.0, and 28 (32.6%) of them were male. Polymyositis (44.2%) was the most common diagnosis, followed by dermatomyositis (43.0%) and juvenile dermatomyositis (12.8%). Hypertension was the most common comorbidity present before the diagnosis of DM/PM, and the anti-Jo-1 antibody was tested and detected in 67 and 20 (29.9%) patients, respectively. Regarding the clinical features of ASA, fever (36.0%) and ILD (26.7%) were the most frequent symptoms present. Concerning patient outcome, during the median follow-up of 34.2 months, 12 deaths and 11 ICU admissions were observed. Ten out of eleven patients admitted to ICU suffered death, and 7 of 12 deaths (58.3%) occurred within 3 months from DM/PM diagnosis. Among 58 patients who were followed up for more than 1 year, 33 (56.9%) achieved disease remission (Table 1).

A comparison of features of ASA according to anti-Jo-1 antibody positivity and negativity showed that patients with the anti-Jo-1 antibody tended to have ILD more frequently than those without it, although statistical significance was not evident (50.0% versus [vs.] 25.5%, *p* = 0.053) (Table 2).

### 3.2. Clinical Outcomes Based on the Presence of Fever, Interstitial Lung Disease and Anti-Jo-1 Antibody

**Figure 2:**
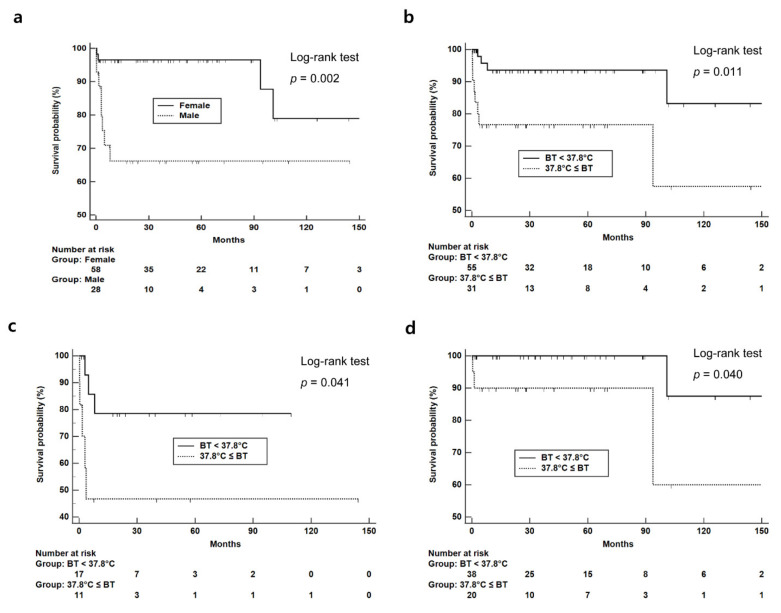
Kaplan–Meier analysis of patient survival based on sex and the presence/absence of fever. The overall survival rate was compared in (**a**) male vs. female patients and in (**b**) total patients, (**c**) male patients, and (**d**) female patients based on the presence and absence of fever. BT: Body temperature.

Given that fever and ILD are associated with patient prognosis in AIDs and that the anti-Jo-1 antibody is associated with severe features in ASA [20,21], we compared the clinical outcomes of the patients according to fever, ILD, and anti-Jo-1 antibody positivity. DM/PM patients with fever had significantly higher mortality than those without (25.8% vs. 7.3%, *p* = 0.024), and the remission rate at 1 year was higher in DM/PM patients with fever than in those without (82.4% vs. 46.3%, *p* = 0.019) (Table 3). Kaplan–Meier analysis revealed that the overall survival rate was lower in male patients (Figure 2a, *p* = 0.002) and in patients with fever than in those without fever, irrespective of sex (*p* = 0.011 for all patients, *p* = 0.041 and *p* = 0.040 for men and women, respectively) (Figure 2b–d). There was no difference in clinical outcomes according to the remaining ASA features of Raynaud’s phenomenon, mechanic’s hand and arthritis.

Concerning medications, although patients with ILD were more likely to be treated with azathioprine (56.5% vs. 25.4%, *p* = 0.007) and less likely to be treated with methotrexate (30.4% vs. 57.1%, *p* = 0.029) than those without ILD (Table 4), no difference was noted in the medications administered to patients who did and did not die (Table 5).

### 3.3. Clinical and Laboratory Features Associated with All-Cause Mortality

In univariable analysis, older age, male sex, comorbidity with diabetes mellitus, ESR, and fever were associated with increased patient mortality; the presence of ILD was not statistically significant. However, when a multivariable analysis was performed, only male sex (hazard ratio [HR] 5.53, 95% confidence interval [CI] 1.65, 18.49, *p* < 0.01) and fever (HR 4.20, 95% CI 1.26, 14.01, *p* = 0.02) were predictive factors of all-cause mortality during the follow-up (Table 6).

## 4. Discussion

The present study’s results confirmed that ASA clinical features are common in patients with DM and PM, with fever and ILD being the most common features. In addition, the mortality and disease remission rates in this study were 14.0% and 56.9%, respectively, similar to data reported by Bronner et al. and Watanabe et al., respectively [12,22]. Moreover, the comparison of the clinical outcomes according to the presence and absence of ASA clinical features showed that DM/PM patients with fever experienced death more often than those without fever. In particular, in the adjusted analysis of variables comprising age, male sex, fever, ESR and comorbidity with diabetes mellitus, male sex and fever were revealed to be significant factors of all-cause mortality. The observations of our study indicate that fever should be considered as one of the alarm signals in patients with DM/PM and that precaution is required for those with fever.

The anti-Jo-1 antibody (anti-histidyl-tRNA synthetase), which is also classified as a myositis specific autoantibody, is the first antibody that was identified among various anti-ARS antibodies in ASA; anti-ARS antibodies are reported be detected in 20–40% of patients with IIM [23,24]. In the current study, 67 (77.9%) patients were tested for the anti-Jo-1 antibody, and 29.9% of the patients showed anti-Jo-1 positivity, which is in line with previous estimates [25]. Studies have suggested that the anti-Jo-1 antibody is related to a higher probability of having ILD in DM/PM and severe clinical features in ASA [20,21]; nonetheless, the prognostic implications of this antibody are still not well established, owing to the various manifestations of the disease in DM/PM patients and differences in study designs. A previous study by Douglas et al. that evaluated the prognosis of DM/PM patients with ILD showed that there was no survival difference for patients with anti-Jo-1 antibody positivity and negativity [26]. In contrast, it was demonstrated by Aggarwal et al. that patients lacking the anti-Jo-1 antibody had worse survival than those with anti-Jo-1 antibody positivity, which was quite unexpected [27]. Our data showed that patients presenting the anti-Jo-1 antibody exhibited an increased tendency of accompanying ILD, whereas there was no difference in the investigated clinical outcomes between those with anti-Jo-1 antibody positivity and those without it. Even though this finding seems to be mainly affected by the discrepancy of designs across studies, it seems apparent that a better understanding of the role of this antibody is required.

In the univariable Cox proportional analysis, age, which is a traditional risk factor for increased mortality in DM/PM patients, was also linked to all-cause mortality [17]. On the other hand, although ILD tended to be a predictor of patient mortality, its effect was not statistically significant. This could be attributed to differences in the definition of ILD or to the effect of medications to treat DM/PM. Indeed, Fathi et al. revealed that most cases of ILD in DM/PM are non-progressive during immunosuppression [28]. Surprisingly, we found that only male sex and fever independently reflected a higher odd of mortality. While it is increasingly recognised that features of ASA could be present in patients with DM/PM, most studies focused on evaluating the relationship between ILD and patient mortality. Interestingly, a research study analysed factors associated with the aggravation of ASA in anti-Jo-1 antibody-positive patients with ASA; yet, the direct effect of ASA clinical features on patient prognosis was not assessed by Marie et al. [21]. To our knowledge, our study is the first to demonstrate that fever is a risk factor for all-cause mortality and to propose that careful attention is essential for patients with DM/PM, especially in the early phases of the disease. Importantly, more than half of the deaths occurred within 3 months after diagnosis, and the clinical impact of fever was consistent in both sexes, emphasizing our findings. However, patients with fever had a higher probability of achieving remission during the first year after treatment when they were alive. Collectively, we believe that this finding provides evidence that active immunosuppression may be beneficial for improving outcome in such patients. Nonetheless, owing to the retrospective study design and the possibility of immortal bias, a cautious interpretation of our data is required.

Although the clear association between fever and increased mortality in DM/PM patients is still uncertain, it is possible that fever may be a surrogate marker indicating higher inflammation, resulting in escalated mortality. This is supported by a previous study demonstrating increased expression of pyrogenic cytokines such as tumour necrosis factor-α, interleukin (IL)-1, IL-6 and interferon-gamma in the circulation and muscles in patients with inflammatory myopathies [29,30], which are also thought to be relevant for disease pathogenesis [31]. Taking into account that fever, rather than conventional laboratory markers of white blood cell and neutrophil counts and ESR, was independently related to patient mortality in this study, fever might be a clinically relevant indicator to identify patients with poor clinical outcomes.

We acknowledge several points that should be considered as limitations of this study. First, this study was conducted retrospectively, which could have affected patient data collection. Second, although there was no difference in the medications that were administered to the patients for disease treatment, the effect of treatment on patient outcomes could not be analysed in detail, owing to the limitations of the retrospective analysis. Third, the presence of myositis-specific antibodies, including anti-melanoma differentiation-associated gene 5 antibody, which is associated with patient prognosis and clinical features of ILD among patients with DM/PM, was not tested in our patients. Fourth, the number of patients included in our analysis was relatively small. Therefore, future large-scale investigations are necessary to validate our result and confirm whether fever is a negative prognostic factor in patients with DM/PM.

## 5. Conclusions

In conclusion, we demonstrated that in patients with DM/PM, male sex and fever were associated with worse overall survival. The results of our study indicate that higher clinical attention and careful monitoring are required for those presenting with fever.

## Figures and Tables

**Table 1:** Baseline data of the 86 patients.

Clinical Characteristics	Values
Demographics	
Age	51.0 (29.0)
Male sex	28 (32.6)
Diagnosis	
Polymyositis	38 (44.2)
Dermatomyositis	37 (43.0)
Juvenile dermatomyositis	11 (12.8)
Previous comorbidities present ^‡^	
Hypertension	19 (22.1)
Diabetes mellitus	13 (15.1)
Dyslipidemia	5 (5.8)
Laboratory results	
White blood cell count (/mm^3^)	7375.0 (3330.0)
Neutrophil count (/mm^3^)	4340.0 (2867.5)
Platelet count (×1000/mm^3^)	274.5 (130.0)
Erythrocyte sedimentation rate (mm/h)	32.0 (34.0)
C-reactive protein (mg/L) (*n* = 81)	2.6 (8.1)
Aspartate aminotransferase (IU/L)	115.0 (192.0)
Alanine aminotransferase (IU/L)	88.5 (144.0)
Creatinine kinase (IU/L)	1884.0 (6684.0)
Anti-Jo-1 antibody positivity (*n* = 67) ^†^	20 (29.9)
Clinical features of anti-synthetase syndrome present	
Fever	31 (36.0)
Raynaud’s phenomenon	9 (10.5)
Mechanic’s hand	5 (5.8)
Arthritis	11 (12.8)
Interstitial lung disease	23 (26.7)
Outcomes	
All-cause mortality	12 (14.0)
Intensive care unit admission	11 (12.8)
Remission at 1 year (*n* = 58) ^¶^	33 (56.9)
Follow-up duration (months)	34.2 (61.1)

Data are presented as median (interquartile range) or number (percentage). ^‡^ The comorbidities investigated included those prior to disease diagnosis. ^†^ Sixty-seven patients were tested for anti-Jo-1 antibody. ^¶^ Fifty-eight patients were followed up for more than 1 year.

**Table 2:** Comparison of clinical features of anti-synthetase syndrome according to anti-Jo-1 antibody positivity and negativity.

	Anti-Jo-1 Antibody (+) Group (*n* = 20)	Anti-Jo-1 Antibody (−) Group (*n* = 47)	*p*-Value
Fever	9 (45.0)	20 (42.6)	0.854
Raynaud’s phenomenon	4 (20.0)	4 (8.5)	0.226
Mechanic’s hand	2 (10.0)	3 (6.4)	0.631
Arthritis	3 (15.0)	7 (14.9)	0.999
Interstitial lung disease	10 (50.0)	12 (25.5)	0.053

Data are presented as percentages.

**Table 3:** Outcomes according to the presence and absence of fever, interstitial lung disease and anti-Jo-1 antibody.

All Patients (*n* = 86)	Fever (*n* = 86)			Interstitial Lung Disease (*n* = 86)			Anti-Jo-1 Antibody (*n* = 67)		
	Yes (*n* = 31)	No (*n* = 55)	*p*-Value	Yes (*n* = 23)	No (*n* = 63)	*p*-Value	Yes (*n* = 20)	No (*n* = 47)	*p*-Value
All-cause mortality (+)	8 (25.8)	4 (7.3)	0.024	6 (26.1)	6 (9.5)	0.051	5 (25.0)	6 (12.8)	0.220
All-cause mortality (−)	23 (74.2)	51 (92.7)		17 (73.9)	57 (90.5)		15 (75.0)	41 (87.2)	
Intensive care unit admission (+)	7 (22.6)	4 (7.3)	0.051	5 (21.7)	6 (9.5)	0.136	5 (25.0)	5 (10.6)	0.134
Intensive care unit admission (−)	24 (77.4)	51 (92.7)		18 (78.3)	57 (90.5)		15 (75.0)	42 (89.4)	
Patients with follow-up ≥1 year (*n* = 58)	Fever (*n* = 58)			Interstitial lung disease (*n* = 58)			Anti-Jo-1 antibody (*n* = 48)		
	Yes (*n* = 17)	No (*n* = 41)	*p*-value	Yes (*n* = 13)	No (*n* = 45)	*p*-value	Yes (*n* = 13)	No (*n* = 35)	*p*-value
Remission at 1 year (+) ^†^ (*n* = 58)	14 (82.4)	19 (46.3)	0.019	5 (38.5)	28 (62.2)	0.131	4 (30.8)	22 (62.9)	0.059
Remission at 1 year (−) ^†^ (*n* = 58)	3 (17.6)	22 (53.7)		8 (61.5)	17 (37.8)		9 (69.2)	13 (37.1)	

Data are presented as percentage. ^†^ Fifty-eight patients were followed up for more than 1 year.

**Table 4:** Medication usage in patients with and without interstitial lung disease.

	Patients with Interstitial Lung Disease (*n* = 23)	Patients without Interstitial Lung Disease (*n* = 63)	*p*-Value
Glucocorticoid	23 (100.0)	63 (100.0)	0.999
Methotrexate	7 (30.4)	36 (57.1)	0.029
Azathioprine	13 (56.5)	16 (25.4)	0.007
Intravenous immunoglobulin	8 (34.8)	19 (30.2)	0.684
Rituximab	1 (4.3)	4 (6.3)	0.999
Hydroxychloroquine	5 (21.7)	20 (31.7)	0.369
Cyclophosphamide	3 (13.0)	5 (7.9)	0.436
Tacrolimus	3 (13.0)	3 (4.8)	0.336
Mycophenolate mofetil	2 (8.7)	4 (6.3)	0.656

Data are presented as number (percentage).

**Table 5:** Medications administered to patients in relation to their death or survival.

	Patients with Mortality (*n* = 12)	Patients without Mortality (*n* = 74)	*p*-Value
Glucocorticoid	12 (100.0)	74 (100.0)	0.999
Methotrexate	3 (25.0)	40 (54.1)	0.117
Azathioprine	3 (25.0)	26 (35.1)	0.743
Intravenous immunoglobulin	5 (41.7)	22 (29.7)	0.411
Rituximab	2 (16.7)	3 (4.1)	0.141
Hydroxychloroquine	2 (16.7)	23 (31.1)	0.496
Cyclophosphamide	2 (16.7)	6 (8.1)	0.309
Tacrolimus	1 (8.3)	5 (6.8)	0.999
Mycophenolate mofetil	1 (8.3)	5 (6.8)	0.999

Data are presented as percentage.

**Table 6:** Cox proportional hazard analysis of factors associated with all-cause mortality.

	Univariable Analysis			Multivariable Analysis		
	HR	95% CI	*p*-Value	HR	95% CI	*p*-Value
Age	1.04	1.01, 1.08	0.02			
Male sex	5.54	1.66, 18.56	<0.01	5.53	1.65, 18.49	<0.01
Hypertension	1.99	0.60, 6.62	0.26			
Diabetes mellitus	3.57	1.07, 11.93	0.04			
Dyslipidemia ^¶^	n/a					
White blood cell count	1.00	1.00, 1.00	0.30			
Neutrophil count	1.00	1.00, 1.00	0.96			
Platelet count	1.00	1.00, 1.00	0.95			
Erythrocyte sedimentation rate	1.02	1.00, 1.03	0.04			
Aspartate aminotransferase	1.00	1.00, 1.00	0.77			
Alanine aminotransferase	1.00	0.99, 1.00	0.37			
Creatinine kinase	1.00	1.00, 1.00	0.68			
Fever	4.20	1.26, 13.97	0.02	4.20	1.26, 14.01	0.02
Raynaud’s phenomenon	0.87	0.11, 6.76	0.90			
Mechanic’s hand ^¶^	n/a					
Arthritis	0.49	0.06, 3.79	0.49			
Interstitial lung disease	3.06	0.98, 9.49	0.05			

n/a: not applicable, HR: hazard ratio, CI: confidence interval. ^¶^ As no event of death occurred in patients with dyslipidaemia and mechanic’s hand, these variables were not included in the Cox proportional hazard analysis.

## Data Availability

The datasets generated during and/or analysed during the current study are available from the corresponding author on reasonable request.
